# Surgical Wound Infections in Plastic Surgery: Simplified, Practical, and Standardized Selection of High-risk Patients

**DOI:** 10.1097/GOX.0000000000002202

**Published:** 2019-04-23

**Authors:** Marta Starnoni, Massimo Pinelli, Giorgio De Santis

**Affiliations:** From the Division of Plastic Surgery, University of Modena and Reggio Emilia, Modena, Italy.

## Sir,

We have found a lot of interest in selecting patients at high risk of surgical wound infection.

Infection at surgical site in plastic surgery can give a suboptimal esthetic outcome, but it can also impair psychosocial well-being, delay hospital discharge, and lead to readmission and further surgery.

In high-risk patients, an improvement in identification, prevention, and management of surgical wound infections is imperative in reducing infections. An implementation of postoperative care is necessary, including close monitoring and assessment, antibiotic prophylaxis, use of nonirritant medical care products, selection of the appropriate disinfectant, and application of specific devices (such as incisional negative pressure wound therapy).

Evaluation of the surgeon that a patient has a “low” or a “high risk” of infection at surgical site may not reflect the real risk, because it depends on surgeon skills. This limitation can be overcome by using a risk index.

Infection Risk Index (IRI), proposed by the National Nosocomial Infections Surveillance,^1^ was developed to predict surgical infection risk of the patient and to compare surgical infection rates among surgeons, among institutions, or across time. IRI consists of 3 risk factors: (1) A patient having an American Society of Anesthesiologists preoperative assessment score of 3,4, or 5; (2) An operation classified as either contaminated or dirty infected; and (3) An operation with duration of surgery more than T hours, where T depends on the operative procedure being performed. Surgical infection rate increases from patients with none of the risk factors, to patients with all the 3 risks.

We asked 2 inexperienced surgeons (couple A) and 2 experienced surgeons (couple B) to select patients with high risk of surgical wound infection according to their opinion, in a group of 100 patients undergoing surgery (20 breast reconstructions, 20 postbariatric procedures, 20 scar revisions, 20 cancer removals, 20 debridement and skin grafting). We compared their opinion with IRI calculation. Patients with 2 or 3 risk factors (IRI 2–3) were considered to be at high risk of infection. We recorded a corresponding result between surgeon opinion of “high risk” and IRI 2–3 of 64% in couple A and of 94% in couple B.

IRI is feasible, readily available at the end of surgery, and can perform well across a broad range of operative procedures (plastic surgery ranges from cosmetic to reconstructive procedures of all body parts). The traditional wound classification system (clean, clean-contaminated, contaminated, dirty infected) has important limitations (such as its failure to account for intrinsic patient risk) that can be overcome considering American Society of Anesthesiologists score (index of intrinsic host susceptibility) and operation time (marker for the complexity of the individual surgical technique and for the possible reduction of the effect of antibiotic prophylaxis).

In our opinion, IRI can be used routinely for its simplicity and can be considered better than surgeon opinion, especially in inexperienced surgeons. It is not specific for plastic surgery but it allow surgeons working in the same department (but with different experience and opinion) to standardize selection of high-risk patients undergoing different surgical procedures.
